# An *in-vitro* animal experiment on metal implants’ thermal effect on radiofrequency ablation

**DOI:** 10.1186/1477-7819-11-147

**Published:** 2013-06-22

**Authors:** Zhen-Wen Lin, Hong Chu, Fan He, Lu-Ping Wang, Jian Kong

**Affiliations:** 1 Minimally Invasive Interventional Treatment Center, Shenzhen People’s Hospital, Shenzhen, Guangdong, People’s Republic of China; 2 Department of Gynecology, Shenzhen Second People’s Hospital, Shenzhen, 3002 Sungang West Road, Futian district, Shenzhen, Guangdong 518035, People’s Republic of China

**Keywords:** Implant, Metal, Radiofrequency ablation, Thermal effect, Animal experiment, In vitro

## Abstract

**Background:**

To explore metal implants’ thermal effect on radiofrequency ablation (RFA) and ascertain distance-thermal relationship between the metal implants and radiofrequency (RF) electrode.

**Methods:**

Metal implants models were established in seven *in-vitro* porcine livers using silver clips or ^125^I seeds. RFA were conducted centering the RF electrode axis1 cm away from them, with one side containing a metal implants model the test group and the other side the control group. The thermometric needles were used to measure multi-point temperatures in order to compare the time-distance-temperature difference between the two groups. The gross scopes of the ablation of the two groups were measured and the tissues were analyzed for microscopic histology.

**Results:**

At the ablation times of 8, 12, and 15 min, the average multi-point temperatures of the test group and the control group were 48.2±18.07°C, 51.5±19.57°C, 54.6±19.75°C, and 48.6±17.69°C, 52.2±19.73°C, 54.9±19.24°C, respectively, and the differences were not statistically significant (*n*=126, *P*>0.05). At the ablation times of 12 and 15 min, the ablation scopes of the test group and the control group were (horizontal/longitudinal diameter) 1.55/3.48 cm, 1.89/3.72 cm, and 1.56/3.48 cm, 1.89/3.72 cm, respectively, and the differences were not statistically significant (*n*=14, *P*>0.05). The two groups had the same manifestations in microscopy.

**Conclusions:**

Metal implants do not cause significant thermal effect on RFA.

## Background

Radiofrequency ablation (RFA) is a common and effective interventional treatment for solid tumors [1]. In recent years, with the rapid development of interventional treatment and availability of multidisciplinary remedies, many cancer patients may have received some metal implants (such as silver clips, titanium clips, ^125^I seeds) delivered during hepatocarcinoma resection or interstitial ^125^I seed implantation before undergoing the RFA, raising concerns as whether these metal implants impact on the radiofrequency (RF) or cause thermal hurt to the neighboring blood vessels or bile ducts. In this experiment, silver clips and ^125^I seeds were implanted in porcine livers *in vitro* and a cuboid array of metal implants model was established to explore the metal implants’ thermal effect on RFA.

### Materials and methods

#### Materials

Seven fresh and complete porcine livers with the maximum thickness >40 mm, 54 silver clips (Baote Shanghai, China) and 54 ^125^I seeds (Atomic Hi-Tech, China) were needed. The ^125^I seed consisted of an outer titanium capsule of 0.05 mm in thickness, 4.5 mm long, and 0.8 mm in diameter, containing ^125^I adsorbed onto a 3.0 × 0.5 mm silver rod. Taking into account radiation protection issues and the purpose of the experiment, the ^125^I seeds used in this experiment were inactive.

#### Equipment

The main equipment comprised Cool-tip™ RF ablation system (CovidienValleylab, Boulder, CO, USA), 17-gaugestraight monopolar Cool-tip™ RF electrode (CovidienValleylab, Boulder, CO, USA), epidural needles (18G × 150 mm, Dr.J, Japan), graduated thermocouple thermometric needles and digital thermometer (NanjingKangyou, China).

## Methods

### Metal implants modeling

**Figure 1:**
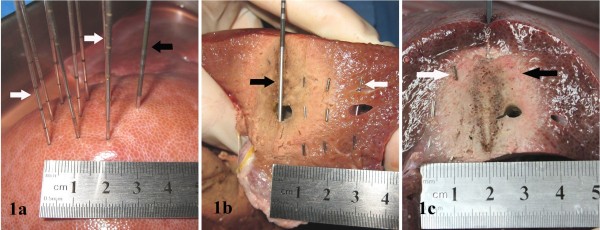
**Modeling and grouping. (a)** vertical multi-pin array for test group. Black arrow: RF electrode. White arrow: nine epidural needles used for the implantation in square array (side length of 2.0 cm). **(b)** Section to reveal metal implants embedded in liver tissue constructing a cuboid. Black arrow: RF electrode, 1.0 cm away from metal implants model. White arrow: test group with ^125^I seeds. **(c)** Section of liver after RFA. White arrow: test group (containing silver clips). Black arrow: control group. The ablation scopes of the both groups had no differences.

A liver was placed flat in a metal tray. In the left or right lobe where the tissue thickness was >40 mm, simulating the clinical procedure of interstitial seeds implantation, nine epidural needles (18G, Dr.J) were vertically inserted 3.5 cm deep from the liver surface with a gap of 1.0 cm for each other, forming a square with a side length of 2.0 cm (Figure 1a). After insertion the core of needle was withdrawn and a silver clip or a seed was manually pushed in from the needle tail by a blunt push rod. Subsequently the second and the third silver clip or seed were implanted by the same approach by withdrawing the needle 1.0 cm each time. Each model accommodated 27 implants constructing a cuboid of 2.0 × 2.0 (bottom) × 3.5 cm (height) (Figure 1b). The silver clips and ^125^I seeds were reused, namely, the 27 metal implants were used by the models one by one after collection.

### RFA procedure

Two grounding pads were affixed to the bottom of the metal tray. RF electrode was vertically inserted 4.0 cm deep from the liver surface and 1.0 cm away from the metal implants model (Figure 1a and 1b). Then the peristaltic pump and RF generator were connected with impedance mode. The total ablation time was 15 min. Multi-point thermometry was conducted at the ablation times of 8, 12, and 15 min, respectively.

### Grouping

Centering the RF electrode axis, the side accommodating the metal implants was the test group, and the opposite side with only the normal liver tissue the control group (Figure 1b and 1c).

### Thermometry

**Figure 2:**
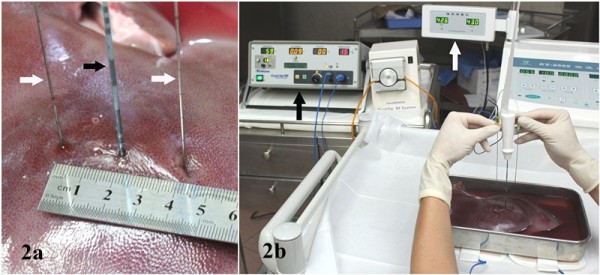
**Symmetrical thermometry in RFA. (a)** two thermometric needles were symmetrically inserted for thermometry centering RF electrode. Black arrow: RF electrode. White arrow: graduated thermometric needles, one for test group, the other for control group. **(b)** a panorama of thermometry in RFA. White arrow: digital thermometer. Black arrow: cool-tip RF generator.

To avoid interference caused by the thermometric needles, thermometric needles were inserted into the liver for timed thermometry and were withdrawn immediately after that. Each thermometric needle was connected to a digital reading panel displaying real-time needle tip temperature with an accuracy of 0.1°C (Figure 2). During the RFA, intermittent and symmetric and multi-point thermometry was conducted at 8, 12, and 15 min by inserting the thermometric needles at three sites of 1.0 cm, 2.0 cm, and 3.0 cm away from the RF electrode in vertical distance, respectively. The depth of the needle tip for each site was 4.0 cm, 3.0 cm, and 2.0 cm in turn by withdrawing. Centering the RF electrode axis, the tissue temperatures were measured simultaneously and symmetrically for both sides, namely test group and control group (Figure 2).

### Ablation scope measurement

Twelve and 15 minutes after RFA, along the RF electrode and the surface accommodating the thermometry points, the liver was sectioned into two halves measuring the ablation scopes of the both groups. The demarcation was defined by naked eyes as the line between the gray coagulation tissue and normal dark red tissue. Ablation hemispheres on both sides along RF electrode were measured by recording the horizontal/longitudinal diameter. Meanwhile, histology examination was conducted for the tissues at the thermometry points and along the demarcation. The specimens were then fixed in 10% formalin for routine histologic processing and were finally processed with paraffin sectioning and hematoxylin-eosin staining for light microscopic study.

### Statistical analysis

The SPSS 13.0 software was used for statistical analysis comparing differences in temperature and ablation scope between the test and control groups at different ablation time and different distances away from RF electrode. The paired-samples t test and independent-samples t test were used to compare the means and *P*<0.05 was defined statistically significant.

## Results

### Metal implants modeling

In total 14 *in-vitro* silver clip models and 14 *in-vitro*^125^I seed models were established, meeting the design requirements confirmed by gross anatomy (Figure 1b and 1c).

### Intraoperative thermometry

In total 14 serial thermometries were conducted, including nine points for thermometry in each group per case and thus 126 values of thermometry in total for each group. When the ablation time was 8, 12, and 15 min, the average multi-point temperatures at different distances of the test group and the control group were 48.2±18.07°C, 51.5±19.57°C, and 54.6±19.75°C, and 48.6±17.69°C, 52.2±19.73°C, and 54.9±19.24°C, respectively. The temperature differences at the individual ablation times were not statistically significant (*n*=126, *P*>0.05) (Table 1). The same was for the stratified analysis (*P*>0.05) (Table 2). At the distance of 1.0 cm away from RF electrode, the average temperatures of the test group and the control group were 71.7±6.79°C, 78.0±4.81°C, and 81.3±5.32°C, and 71.1±7.84°C, 78.9±5.13°C, and 80.9±5.02°C according to the ablation time of 8, 12, and 15 min, respectively (*n*=42, *P*>0.05). Correspondingly, at the distance of 2.0 cm, the temperatures were 43.3±2.55°C, 43.3±1.84°C, and 46.1±3.50°C, and 44.2±3.89°C, 44.2±3.20°C, and 47.1±2.83°C, respectively (*n*=42, *P*>0.05). And at the distance of 3.0 cm, the temperatures were 29.8±2.16°C, 33.1±2.58°C, and 36.5±2.48°C, and 30.5±1.98°C, 33.7±2.32°C, and 36.8±2.01°C, respectively (*n*=42, *P*>0.05) (Table 2).

**Table 1:** Comparison of average multi-point temperatures at different ablation times of test group and control group

**Ablation time (min)**	**Case number**	**Thermometry site (*n *)**	**Average multi-point temperature of test group (**$\bar{x}\pm s$**, °C)**	**Average multi-point temperature of control group (**$\bar{x}\pm s$**, °C)**	**Independent-samples t test**
8	14	126	48.2±18.07	48.6±17.69	*P*>0.05
12	14	126	51.5±19.57	52.2±19.73	*P*>0.05
15	14	126	54.6±19.75	54.9±19.24	*P*>0.05

**Table 2:** Stratified analysis for distance-time-temperature relationship between the two groups

**Distance from RF electrode (cm)**	**Case number**	**Therm ometry site ( *n *)**	**Temperature of test group (**$\bar{x}\pm s$**, °C)**			**Temperature of control group (**$\bar{x}\pm s$**, °C)**			**Paired- samples t test**
			**Ablation 8 min**	**Ablation 12 min**	**Ablation 15 min**	**Ablation 8 min**	**Ablation 12 min**	**Ablation 15 min**	
1.0	14	42	71.7±6.79	78.0±4.81	81.3±5.32	71.1±7.84	78.9±5.13	80.9±5.02	*P*>0.05
2.0	14	42	43.3±2.55	43.3±1.84	46.1±3.50	44.2±3.89	44.2±3.20	47.1±2.83	*P*>0.05
3.0	14	42	29.8±2.16	33.1±2.58	36.5±2.48	30.5±1.98	33.7±2.32	36.8±2.01	*P*>0.05

### Ablation scope in gross observation

At ablation times of 12 and 15 min, both groups formed regular coagulation hemispheres, with the test group 1.55±0.122/3.48±0.112 cm (horizontal/longitudinal diameter), 1.89±0.198/3.72±0.163 cm; and the control group 1.56±0.122/3.48±0.112 cm, 1.89±0.207/3.72±0.163 cm (Figure 1c). The differences were not statistically significant (*n*=14, *P*>0.05) (Table 3).

**Table 3:** Comparison of ablation scopes of the two groups

**Ablation time (min)**	**Case number**	**Ablation scope of the test group (**$\bar{x}\pm s$**, cm)**		**Ablation scope of the control group (**$\bar{x}\pm s$**, cm)**		**Independent-samples t test**
		**Horizontal diameter**	**Longitudinal diameter**	**Horizontal diameter**	**Longitudinal diameter**	
12	14	1.55±0.122	3.48±0.112	1.56±0.122	3.48±0.112	*P*>0.05
15	14	1.89±0.198	3.72±0.163	1.89±0.207	3.72±0.163	*P*>0.05

### Histopathologic manifestations under light microscope

The symmetric points of the both groups had the same manifestations under light microscope. The central necrosis area was of coagulation necrosis, showing mass uniform red dye substances, disappeared hepatocyte and lobular architecture, and damaged hepatic cord. The ablation border was of pink edema belt presenting atypical necrosis, dilated sinusoidal, atrophy of residual liver cells, blurred or condensed nuclei. The structure outside the congestive belt was normal.

## Discussion

RFA causes coagulation necrosis of the tumor lesions by inserting the RF electrode into the tumor, which tip generates an RF electric field resulting in the ion-friction thermal effect and temperature increasing in the target area up to 50°C to 100°C. The cool-tip RFA can effectively alleviate charring and gasification around the tip, enlarging the ablation range and widening the clinical application. The RFA time for solid tumors is usually 12 to 15 min. When a monopolar electrode is used, the ablation scope *in vivo* is approximately a 3 cm diameter sphere;while *in vitro*, it can reach a diameter sphere of nearly 4 cm due to absence of blood flow [2]. Tumor patients eligible for RFA may have received various metal implants such as stents, silver clips, titanium clips, or ^125^I seeds left over after the surgery, which are often adjacent to important blood vessels, bile ducts, or intestines, raising concerns about thermal damage [3,4], and urging experimental data for assessment.

This study established the titanium and silver implant models in porcine livers simulating residuals after hepatocarcinoma resection and ^125^I seeds implantation. The parallel needle array and seed release technology enabled accurate and controllable implantation for metal implant modeling in this study. Setting the test and control groups symmetrically on both sides of the RF electrode guarantees objective reflection of the real-time temperature changes and ablation scope. Besides, on the issue of choosing ablation time, thermometry was conducted at points of ablation time from 1 to 15 min in the previous study and all the thermometry results showed no statistical significance was seen in both groups, so three representative time points, namely, 8, 12,and 15 min which were clinically in common use were finally chosen and reported in the study.

According to previous studies [5,6], multi-lead thermocouple thermometry is widely used as the most popular method for thermometry, which inserts the thermometric needles or lines into the tissue measuring real-time temperatures. However, the persistent retention of the metal needles or lines may interfere in the RF electric field [7]. This study intends to assess in-tissue metal implants’ thermal impact on RFA, so a method of intermittent and symmetrical thermometry which inserts needles when thermometry is needed and withdraws needles immediately when thermometry is done, instead of retaining thermometric needles or lines in tissue was applied to reducing the potential metal interference. In addition, the power of RFA automatically fluctuates according to the changes of tissue impedance and results in subtle changes of in-tissue temperature. Therefore, it is necessary to measure the temperatures symmetrically on both sides around the RF electrode, namely, the test and control group, to ensure accuracy. Nonetheless, there are some flaws for this method in that it cannot avoid the slight errors in distances when repeating the puncture, and it is difficult to conduct multi-pin or multi-point puncture at each time. Therefore, this experiment applied one-person double-pin puncture to ensure accuracy and stable operation.

The results of this study showed that the thermometry results of the test group and control group determined on symmetric points with equal distance from the RF electrode at different ablation times had no significant difference, nor the ablation scope confirmed by gross measurement and microscopy, indicating the metal implants will not significantly affect the thermal field of RFA. It is believed by the authors that this is due to the mechanism of RF generating. In the RF circuit, electric current enters through both the RF electrode and the grounding pad which is affixed onto the metal tray with the porcine liver as resistor. Being embedded in the liver, the metal implants donot directly contact with the metal tray or the RF electrode, so they cause little effect on electric current as well as thermal field. But a study [8] reported that Chiba needle once caused thermal hurt to the human skin during RFA, it is speculated due to the naked needle tail which directly contacts the human skin forming a circuit. This phenomenon is still worthy of further in-depth study.

## Conclusions

The preliminary result of this experiment suggests that the presence of metal implants in the target tumor ablation area will not undermine the safety of RFA treatment, nor significantly affect the ablation scope. Many extant studies have revealed that RFA take nearly the same effect *in vivo* as *in vitro*, so it can be predicted that the same scenario as in this experiment will appear *in vivo*. Meanwhile, it is suggested by this experiment that with the presence of metal implants the thermal hurt to the non-target area could be avoided only if the RF electrode does not directly contact the metal implants and regular gap is reserved between the electrode and the non-target area. Yet it needs electrophysiological study and organelle study by electron microscope to make this conclusion more objective. Further studies still need to be conducted in the future.

## Abbreviations

RF: Radiofrequency; RFA: Radiofrequency ablation.

## Competing interests

There is no conflict of interests and no financial benefit has been obtained from any company.

## Authors’ contributions

Z-WL designed and carried out the study, participated in the sequence alignment. HC drafted the manuscript and participated in the sequence alignment. FH, L-PW, and JK participated in the experiment and performed the statistical analysis. All authors read and approved the final manuscript.
